# The Sub-Saharan Africa Regional Partnership (SHARP) for Mental Health Capacity Building: a program protocol for building implementation science and mental health research and policymaking capacity in Malawi and Tanzania

**DOI:** 10.1186/s13033-019-0327-2

**Published:** 2019-11-09

**Authors:** Christopher Fittipaldi Akiba, Vivian Go, Victor Mwapasa, Mina Hosseinipour, Bradley Neil Gaynes, Alemayehu Amberbir, Michael Udedi, Brian Wells Pence

**Affiliations:** 1 363 Rosenau Hall, CB# 7440, Chapel Hill, NC 27599 USA; 2 Centre for Reproductive Health, Malawi College of Medicine, P/Bag 360, Chichiri, Blantyre 3, Malawi; 3 UNC Project, Tidziwe Centre, Private Bag A-104, Lilongwe, Malawi; 4 101 Manning Drive First Floor, Chapel Hill, NC 27514 USA; 5 27 King’s College Circle, Toronto, ON M5S 1A1 Canada; 6 Ministry of Health, Malawi, P.O. Box 30377, Lilongwe, Malawi; 7 2103C McGavran-Greenberg Hall, CB #7435, Chapel Hill, NC 27599 USA

**Keywords:** Mental health, Global health, Capacity building, Protocol, Malawi, Tanzania

## Abstract

**Background:**

Mental health (MH) disorders in low and middle-income countries (LMICs) account for a large proportion of disease burden. While efficacious treatments exist, only 10% of those in need are able to access care. This treatment gap is fueled by structural determinants including inadequate resource allocation and prioritization, both rooted in a lack of research and policy capacity. The goal of the Sub-Saharan Africa Regional Partnership for Mental Health Capacity Building (SHARP), based in Malawi and Tanzania, is to address those research and policy-based determinants.

**Methods:**

SHARP aims to (1) build implementation science skills and expertise among Malawian and Tanzanian researchers in the area of mental health; (2) ensure that Malawian and Tanzanian policymakers and providers have the knowledge and skills to effectively apply research findings on evidence-based mental health programs to routine practice; and (3) strengthen dialogue between researchers, policymakers, and providers leading to efficient and sustainable scale-up of mental health services in Malawi and Tanzania. SHARP comprises five capacity building components: introductory and advanced short courses, a multifaceted dialogue, on-the-job training, pilot grants, and “mentor the mentors” courses.

**Discussion:**

Program evaluation includes measuring dose delivered and received, participant knowledge and satisfaction, as well as academic output (e.g., conference posters or presentations, manuscript submissions, grant applications). The SHARP Capacity Building Program aims to make a meaningful contribution in pursuit of a model of capacity building that could be replicated in other LMICs. If impactful, the SHARP Capacity Building Program could increase the knowledge, skills, and mentorship capabilities of researchers, policymakers, and providers regarding effective scale up of evidence-based MH treatment.

## Background

### Protocol version 1.0 9/12/2017

Mental health disorders, including depressive disorders, anxiety disorders, schizophrenia, and bi-polar disorder, account for nearly one-third of years lived with disability (YLDs), making them the leading cause of YLDs and the fifth leading cause of disability adjusted life years (DALYs) globally [1, 2]. Individuals affected by mental health disorders are at increased risk of suicide as well as cardiovascular, cerebrovascular, and respiratory diseases, resulting in a two to threefold increase in the overall mortality rate [3–5].

In low and middle-income countries (LMICs), mental health disorders account for nearly the same disease burden (11.3% of DALYs) as HIV/AIDS (13%) [6, 7]. In certain key populations, such as children and adolescents or those living with HIV, the burden of depressive disorders is two to five times higher in sub-Saharan Africa compared to those in high-income countries (HICs) [8–10]. This burden is likely exacerbated due to a lack of research, well trained human resources, and government leadership regarding mental health. Mental health publications make up just 3–4% of the overall health literature, and only 6% of mental health publications originate from LMICs [11]. Eaton et al. [12] found that 40% of individuals in LMIC government leadership roles identified poor awareness, and low priority or poor commitment at the government level, as major barriers to development of mental health services with one Nigerian official stating that “[There is a] lack of political will to provide a workable mental health policy, introduce reforms in health service delivery, and poor funding at all levels of government [12].”

Recent systematic reviews and meta-analyses confirm efficacy of mental health treatments. Treatments delivered by non-specialized health workers have reduced perinatal depression, alcohol use disorder, and PTSD symptoms [13]. In addition, school-based mental health interventions significantly increased students’ self-esteem, motivation, and coping skills [14]. Social contact interventions have improved stigma-related mental health knowledge and attitudes [15]. While promising, scale up of these interventions has been limited, underscoring the need to build capacity in implementation science and mental health in LMICs [16].

Limited scale-up of evidence-based mental health interventions are largely due to a lack of policy action and treatment capacity. The World Health Organization (WHO) considers government spending in LMICs for mental healthcare to be very low (less than USD $2 per capita), with the majority of spending focused on treating only the most severe cases [17]. In terms of treatment capacity, the rate of mental health workers is just 1 per 100,000 population in LMICS, a rate 50 times greater in HICs [17].

Available data show the prevalence of depression and other common mental disorders (across primary care and specialty care settings) in Malawi and Tanzania to range between 10.7–30.4% [18–20] and 33.8–78.2% respectively [21–23].

In addition to high prevalence of mental health disorders, 23 of the 46 nations that comprise sub-Saharan Africa are designated as “low-income” status, including Malawi and Tanzania [24]. In addition to economic challenges, Malawi and Tanzania allocate relatively small percentages of their healthcare budgets to mental health, just 1% and 2.4% respectively [25]. While both countries have legislation specific to mental health, the World Health Organization assigned the lowest possible rating for the extent to which Malawi’s laws were in line with human rights covenants (ratings are not available for Tanzania) [26]. Hennink and Stephenson [27] examined the interface among researchers and policymakers in both Malawi and Tanzania. Their work uncovered barriers including researchers’ frustrations that policymakers do not value empirical evidence, and policymakers’ claims that researchers do not effectively disseminate their findings [27]; the authors concluded that collaborative strategies, like collaborative research, are crucial in increasing the uptake of research evidence into health policy [27].

Launched in September of 2017, the Sub-Saharan Africa Regional Partnership (SHARP) for Mental Health Capacity Building (NIMH Project # U19MH113202-01) addresses this lack of research, treatment, and policy capacity through several components described in this protocol. In their review of clinical trial protocols, Chan et al. [28] describe the key role protocols play in describing the planning, conduct, interpretation, oversight, and review of a research study. Protocols provide authors with an opportunity to describe the background, rationale, and methodology of their trials in greater detail than normally afforded in an outcome evaluation [29]. While SHARP’s capacity building program is not a trial, it is a set of components designed to build skills and knowledge with the distal goal of increasing implementation science and mental health research and policy capacity in Malawi and Tanzania. The last two decades have seen an acceleration of capacity building research across a range of health domains [30–34]. While authors often conclude positive results of their capacity building efforts, to our knowledge, a protocol that describes a capacity building program’s components in detail has not been published. Similar to how trial protocols provide a space for interventionists to expand upon their background, rationale, and methodology, this paper aims to explain SHARP’s capacity building program in similar detail. While this protocol does include some preliminary results, they are intended to provide the reader with an idea of implementation thus far rather than acting as a substantive outcome evaluation, which will follow at the conclusion of the 5-year study period.

This capacity building protocol specifically describes: (1) an overview and rationale for the SHARP Capacity Building Program’s five core activities: introductory and advanced short courses, a multifaceted dialogue, on the job training, pilot grants, and “mentor the mentors” courses aimed at building the capacity of mental health researchers, policymakers, and providers in Malawi and Tanzania, (2) the protocol for delivering and evaluating these components, (3) preliminary results, and (4) a discussion on the implications of the delivered components.

In 2013, 194 of the WHO’s member states adopted the Mental Health Action Plan 2013–2020 [35]. The plan outlines four objectives (1. Strengthening effective leadership and governance for mental health; 2. Providing comprehensive, integrated and responsive mental health services in the community; 3. Implementing promotion and prevention strategies; and 4. Strengthening information systems, evidence and research for mental health) reliant across six “cross-cutting principles and approaches” (1. Universal health coverage; 2. Human rights; 3. Evidence-based practice; 4. Life course approach; 5. Multisectoral approach; and 6. Empowerment of persons with mental disorders and psychosocial disabilities) [35]. The SHARP Capacity Building Program aligns with the Mental Health Action Plan 2013–2020 in several ways. In terms of universal health coverage, a study by Leslie et al. [36] found that Malawi and Tanzania have high and moderate coverage of primary care services (81.2% and 67.5% respectively). However, Schmidt et al. [37] caution that universal coverage alone not be used as a marker of success given inherent risks in expanded health services resulting in poorer health status and increased inequality overall. While influencing country level economics and health system coverage fall outside the scope SHARP’s Capacity Building Program, we propose to work within existing health, education, and government systems to strengthen the capacity of the actors within. Schmidt et al. [37] discuss the strengthening of health workforces in achieving effective and responsible universal health coverage. More specifically that “[capacity building] should promote the public health workforce development and be implemented in ways that reduce brain-drain likelihood [37].” SHARP expands such capacity building efforts beyond the health workforce and targets Malawian and Tanzanian mental health researchers, NGO workers, and policymakers. The description of program components below describe further alignment with the Mental Health Action Plan 2013–2020 by strengthening leadership and governance, evidence, and research for mental health in Malawi and Tanzania.

## Methods

### Overview of program

The SHARP Capacity Building Program aims to (1) build implementation science skills and expertise among Malawian and Tanzanian researchers in the area of mental health; (2) ensure that Malawian and Tanzanian policymakers and providers have the knowledge and skills to effectively apply research findings on evidence-based mental health programs to routine practice; and (3) strengthen dialogue between researchers, policymakers, and providers leading to efficient and sustainable scale-up of mental health services in Malawi and Tanzania over a 5 year period (see Table 1). SHARP activities are delivered in partnership between the University of North Carolina at Chapel Hill, the Malawi Ministry of Health, UNC-Project Malawi, Dignitas International, the Malawi Epidemiology and Intervention Research Unit (MEIRU), the University of Malawi College of Medicine, the Tanzania Ministry of Health, and Muhimbili University of Health and Allied Services (MUHAS). Our program builds on many longstanding relationships involving health research, capacity building and service delivery in other health disciplines, particularly HIV infection, including the University of Malawi College of Medicine, UNC-project Malawi (with its Fogarty International Center HIV research training programs) and the current Malawi HIV implementation science research training program (M-HIRST). These programs provided a framework for implementation science short courses, internships, and small grant mechanisms that informed the design of the SHARP capacity-building program. While SHARP is one of 10 global mental health hubs funded by a grant from the United States Government’s National Institute of Mental Health, its implementation relies heavily on the contribution from Malawian and Tanzanian partners in both Ministries of Health, academic institutions, and non-government organizations. Discussed in more detail in the sections below, the vast majority of those implementing SHARP components are Malawians and Tanzanians with the goal of building their capacity to continue delivering activities after the conclusion of the grant cycle.

**Table 1:** SHARP timeline of activities

Program component	Target audience	Target # participants during each year				
		Year 1	Year 2	Year 3	Year 4	Year 5
Introductory short courses (5 days in same week)						
a.m.—implementation science and mental health p.m.—evidence-based mental health interventions	Researchers, policymakers, providers	20 (Cohort 1)	20 (Cohort 2)		20 (Cohort 3)	
Advanced short courses (5 days in same week)						
a.m.—grant writing p.m.—translating research to practice	Researchers, policymakers, providers			10 (Chosen from Cohorts 1 and 2)		10 (Chosen from Cohorts 1–3)
Multifaceted dialogue						
Journal clubs (4/year)	All SHARP affiliates	80	80	80	80	80
Webinars (2/year)	All SHARP affiliates	40	40	40	40	40
Community forum (ongoing)	All SHARP affiliates	20	40	40	60	60
On-the-job training	Researchers, providers		1–2	1–2	1–2	1–2
Pilot grants	All SHARP affiliates	3–5 funded grants	3–5 funded grants	3–5 funded grants	3–5 funded grants	3–5 funded grants
Mentor the mentors short courses (2 days in same week)				Subset of 10 (chosen from Cohorts 1 and 2)		Subset of 10 (chosen from Cohorts 1 and 2)

### Participants

SHARP will recruit three introductory short course cohorts of 20 researchers, policymakers, and providers from Malawi and Tanzania during years 1, 2, and 4 of the program. Eligible participants will hold current positions in Malawian or Tanzanian research institutes, government ministries, or NGOs and have an interest and ability in working to improve mental health outcomes in those settings. Calls for applications will be sent out through partner organization listservs, direct emails, local academic conferences, and academic courses in March of years 1, 2, and 4 of the SHARP Capacity Building Program. Applications will be scored by a panel of reviewers comprising SHARP investigators, co-investigators, and stakeholders based on the strength and clarity of the participants’ statement of interest, how the applicant proposes to use knowledge and skills gained through the courses, and their plan for local mentorship. Once admitted, introductory short course cohort members will have the opportunity to join the introductory short courses, journal clubs, webinars, and the community forum. The cohort will also be invited to apply for the pilot grants and senior traineeship (which could include the advanced short courses, the on-the-job training program, and/or the “mentor the mentors” program). Each cohort will also be invited to join the community forum.

### Capacity building components

#### Short courses

To address the gap in mental health research and research-informed policymaking in LMICs, the SHARP Capacity Building Program will provide short courses to at least 36 Malawian and 24 Tanzanian researchers, policymakers, and providers. Cohort 1 will include 16 Malawians and 4 Tanzanians, Cohort 2 will include 16 Tanzanians and 4 Malawians, and Cohort 3 will include 16 Malawians and 4 Tanzanians. In their meta-analysis of continuing education interventions, Mansouri et al. [38] found that multifaceted and interactive small group learning opportunities had a significant effect on provider knowledge. To this end, the short courses of the SHARP Capacity Building Program focus on designing implementation studies on contextually appropriate, evidence-based mental health interventions and on translating research findings into routine practice. The program offers both introductory and advanced short courses. Introductory courses are conducted over 5 days for 4 h each day, and offered in years 1, 2, and 4 of the 5-year study period. Advanced courses are delivered in years 3 and 5 pulling the most engaged participants, or senior trainees, from years 1–2, and 4 respectively.

Introductory courses focus on implementation science research methods as well as evidence-based mental health interventions. The first course, implementation science and mental health, introduces implementation science basics, theories and conceptual models for identifying barriers and facilitators to scale-up, implementation strategies for mental health interventions, and design and evaluation of implementation science studies in mental health. The second course, evidence-based mental health interventions, presents the concept of an evidence base and how much evidence is needed for scale-up from a research and policy perspective. The course also reviews the evidence base for a range of mental health interventions in LMICs, and discusses how to select culturally and contextually appropriate interventions for a given setting. These in-person courses offer the opportunity for researchers, policymakers, and providers from both Malawi and Tanzania to participate in discussions and activities regarding mental health interventions, scale-up, and policies.

Participants from the introductory short course cohorts will be invited to apply to the two advanced short courses in years 3 and 5. The SHARP team will select up to 10 top candidates, or senior trainees, per year offered. Advanced short courses focus on grant writing and translating research into practice. The grant writing course is designed for senior trainees with an identified grant deadline (including SHARP pilot grant submissions; discussed below) and is a practical program to “jumpstart” the grant submission. It focuses on implementation science grants and areas that are typically challenging for new investigators: specific aims, implementation science conceptual frameworks, study designs to evaluate implementation interventions, and implementation outcomes. Trainees will work on their proposals, review each other’s proposals, and receive one-on-one mentoring during the week. The second advanced course, translating research into practice, focuses on policymakers’ ability to interpret research manuscripts, select culturally and contextually appropriate evidence-based interventions for mental health, using research findings to inform mental health policies, and the detailed pathways through which government policy is determined. The course uses specific examples of policies informed by scientific research and provide the opportunity through in-class exercises to simulate the translation of a research finding of their choice into policy.

#### Multifaceted dialogue

SHARP will further develop research and treatment capacity by providing a platform of various modalities to facilitate dialogue between researchers, policymakers, and providers to foster translation of mental health research into practice through programs and policies. The platform will comprise an annual mental health conference with the University of Malawi College of Medicine (COM), quarterly journal clubs, twice yearly webinars on implementation science and mental health, and a SHARP community forum to facilitate networking and discussions among mental health researchers, policymakers, and providers in the region.

##### Mental health conference

The Malawi Mental Health Research and Practice Development Conference is an annual meeting sponsored by COM since 2011. It serves as a venue for the dissemination of research and practice developments in mental health in Malawi and the region. It is a multidisciplinary conference for all cadres working in mental healthcare including nurses, clinical officers, psychologists, psychiatrists, and occupational therapists. As co-sponsor, the SHARP Capacity Building Program will facilitate and financially support the participation of introductory short course cohort members, pilot grantees, on-the-job trainees, and other SHARP affiliated researchers, policymakers, or providers. The conference will provide an opportunity for SHARP pilot grantees and on-the-job trainees to present the findings from their research or mentored experiences, and represents an opportunity to enhance dialogue around scale-up of mental health EBIs among governmental, academic, and non-governmental partners in Malawi and Tanzania.

##### Webinars

In order to reinforce existing implementation science concepts and introduce new ones, SHARP will also host twice-yearly webinars coordinated by SHARP’s capacity building co-directors. Each presentation will feature a relevant expert in the field ending with a 30-min discussion on concrete steps to translate new findings into practice.

##### Journal clubs

SHARP journal clubs will be facilitated quarterly on-site and online, in Lilongwe, Malawi; Blantyre, Malawi; Zomba, Malawi; Karonga, Malawi; and Dar es Salaam, Tanzania; to discuss peer-reviewed manuscripts on evidence-based interventions or implementation science studies. These locations represent various headquarters of SHARP’s partner organizations comprised of academic institutions, medical and public health NGOs, and Ministry of Health offices, hospitals, and clinics. Each characterizes different facets of SHARP’s target audience of researchers, policymakers, and providers across a range of locations. Each partner organization will volunteer on a rotating basis to host a journal club discussion on an implementation science and mental health topic of their choice. Discussions will center on the scientific rigor of the study design, interpretation of findings, determining if an intervention has sufficient evidence for scale-up, and detailing next steps to translate findings into policy. Journal clubs are already underway and enrollment-to-date can be viewed in Table 2.

**Table 2:** SHARP capacity building activities and enrollment

Component	Enrollment totals			Enrollment by position			Enrollment by country	
Short courses	# Targeted	# Applied	# Enrolled	Researcher enrollees	Policymaker enrollees	Provider enrollees	Malawian enrollees	Tanzanian enrollees
Introductory short course Cohort #1	20	37	21	16	1	4	16	5
Introductory short course Cohort #2	20	48	16	9	1	6	4	12
Advanced short course Cohort #1	10	Scheduled for 2020						
Introductory short course Cohort #3	20	Scheduled for 2021						
Advanced short course Cohort #2	10	Scheduled for 2022						
Multi-platform dialogue								
Journal club Q1 2018	20	N/A	9	8	0	1	4	3
Journal club Q2 2018	20	N/A	17	17	0	0	17	0
Journal club Q3 2018	20	N/A	15	15	0	0	15	0
Journal club Q4 2018		N/A	N/A	N/A	N/A	N/A	N/A	N/A
Journal club 2019	20	Scheduled for Q1–Q4 2019						
Journal club 2020	20	Scheduled for Q1–Q4 2020						
Journal club 2021	20	Scheduled for Q1–Q4 2021						
Journal club 2022	20	Scheduled for Q1–Q4 2022						
Webinar 2018 Q3	20	N/A	12	12	0	0	11	1
Webinar 2019 Q1	20	Scheduled for Q1 2019						
Webinar 2019 Q3	20	Scheduled for Q3 2019						
Webinar 2020 Q1	20	Scheduled for Q1 2020						
Webinar 2020 Q3	20	Scheduled for Q3 2020						
Webinar 2021 Q1	20	Scheduled for Q1 2021						
Webinar 2021 Q3	20	Scheduled for Q3 2021						
Webinar 2022 Q1	20	Scheduled for Q1 2022						
Webinar 2022 Q3	20	Scheduled for Q3 2022						
Community forum	60	Scheduled for 2018						
On-the-job training								
On-the-job training 2019	1–2	Scheduled for 2019						
On-the-job training 2020	1–2	Scheduled for 2020						
On-the-job training 2021	1–2	Scheduled for 2021						
On-the-job training 2022	1–2	Scheduled for 2022						
Pilot grants								
Pilot grantee Cohort #1	3–5	10 (teams of 2)	4 (teams of 2)	4	4	0	3 (teams of 2)	1 (team of 2)
Pilot grantee Cohort #2	3–5	Scheduled for 2019						
Pilot grantee Cohort #3	3–5	Scheduled for 2020						
Pilot grantee Cohort #4	3–5	Scheduled for 2021						
Mentor the mentors								
Advanced short course Cohort #1	10	Scheduled for 2020						
Advanced short course Cohort #2	10	Scheduled for 2022						

##### SHARP community forum

The SHARP Capacity Building Program will also have an online platform managed and moderated by SHARP faculty members on a weekly rotation. The Community Forum will encourage discussions and Q&A around new mental health implementation science topics. Topics will include reflections on the most recent journal club or webinar topics, recent relevant journal articles, or topics from recent relevant meetings such as the NIMH/Grand Challenges Canada Global Mental Health Workshop.

#### On-the-job training

The SHARP Capacity Building Program will provide mentored real-world training opportunities within the SHARP scale-up study. The scale-up study, an implementation science trial comparing the success of different blended implementation strategies in supporting the integration of depression treatment into routine medical care in Malawi, runs in parallel to the Capacity Building Program. This trial will provide a unique opportunity for select junior staff or on-the-job trainees to participate in the design, conduct, and analysis of the scale-up study.

Specific placements depend on the trainees’ interests and backgrounds and the needs of the scale-up study. On-the-job trainee experiences will include contributing to the design and execution of (1) the implementation strategies, (2) the implementation outcome assessment, including qualitative interviews, (3) the effectiveness outcome assessment, and (4) the cost-effectiveness assessment.

In year 5, mentors and trainees will jointly complete a report on what the trainee learned from the experience, which the trainee will present to the SHARP faculty and partners.

#### Pilot grants

SHARP will establish a pilot grant program for mentored research addressing implementation science in mental health. A request for proposals (RFP) will be released shortly after each short course in order to engage short course cohorts in the pilot grant program.

Applicants will be required to submit proposals as a team of two co-principal investigators, consisting of one researcher or provider and one policymaker, to facilitate dialogue between the two groups. The goals of the pilot grants are to learn and practice hypothesis generation, study design, grant writing, and evaluation of data in a closely mentored environment while generating preliminary data for future implementation science grants. In their work on mentorship in public health research in LMICs, Cole et al. [39] found that “mentoring networks spanning institutions and countries using multiple virtual and face-to-face methods are a potential avenue for fostering organizational cultures supporting quality mentorship in global health research.” SHARP prioritizes crosscutting research teams and multidisciplinary projects, which will develop and strengthen in-country and regional partnerships. Proposal funding will be based on scientific merit, advancement of trainee career development, demonstration of researcher–policymaker collaboration, and the furthering of SHARP goals.

#### “Mentor the mentors”

Research from disciplines like nursing have historically found mentorship to be fundamental to the learning experience [40]. Recent nursing research shows that mentorship can even impact a trainee’s beliefs of evidence-based practices. Wallen et al. [41] found that exposure to an evidence based practice mentorship program was significantly correlated with nurses’ positive beliefs about implementing evidence based practices. In-line with such findings, SHARP’s “mentor the mentors” program will develop senior trainees admitted to the short course cohorts who can serve as independent mentors to medical and public health students in Malawi and Tanzania. Activities will consist of a short mentoring course in years 3 and 5 for senior trainees. These courses will provide training around how to provide effective mentorship to junior researchers and policymakers interested in implementation science and mental health, including topics like developing implementation science in mental health syllabi for short and long courses, interactive in-class activities, and other pedagogical approaches to engaging and mentor students. Like the advanced short courses, this component follows after the introductory short courses so that the participants can harness their implementation science and mental health knowledge and skills as a building block to support their future mentees. The “mentor the mentors” short course will be supplemented with monthly calls with a SHARP faculty member over 6 months. In addition, senior trainees in the “mentor the mentors” program will have co-instructor responsibilities for SHARP introductory and advanced short courses in years 4 and 5 to reinforce in-class training with application of teaching and mentoring skills.

## Discussion

### Participants

To date, SHARP has recruited introductory short course Cohort #1 and 2. Cohort #1 was comprised of 21 participants (16 researchers, 1 policymaker, and 4 mental health providers; 16 Malawian and 5 Tanzanian) and Cohort #2 comprised of 16 participants (9 researchers, 1 policymaker, and 6 mental health providers). Eliciting applications from policymakers proved the biggest challenge to recruitment for both cohorts given the limited number of Ministry of Health officials in Malawi and Tanzania that focus their work on mental health and implementation science. Future recruitment will broaden the definition of “policymaker” to include a wider array of Ministries, as well as targeting multiple levels of policymakers within each ministry. Increasing efforts to expand recruitment of policymakers will help to raise awareness about mental health and increase dialogue at the Ministry level.

### Assessment of outcomes

Table 3 provides a summary of data collection activities and indicators of the five capacity building components. Below are our outcomes for each program component:

**Table 3:** Description of SHARP capacity building program components

Component	Name	Participants	Activities	Indicators
1	Short course programs	Malawian and Tanzanian researchers, policymakers, and providers	3 introductory courses (implementation science and mental health, evidence-based mental health interventions) 2 advanced courses (grant writing, translating research to practice)	Number of courses Attendance at courses Course evaluations (participant satisfaction) Knowledge (participant knowledge pre-post courses)
2	Multifaceted dialogue platform	All SHARP affiliates	Annual Malawi Mental Health Research and Practice Development Conference Twice yearly webinars Quarterly journal clubs SHARP community platform	Number of participants attending conference Number of participant posters/presentations at conference Number of webinars Attendance at webinars (new and returning participants) Number of journal clubs Attendance at journal clubs (new and returning participants) Number of forum posts Content evaluations (participant satisfaction)
3	On-the-job training	Malawian and Tanzanian researchers, policymakers, and providers	On-the-job research training in partnership with SHARP scale-up study	Number of applications for “On-the-job” training program Number of participants for “On-the-job” training program Number of scientific posters/presentations Number of scientific publications
4	Pilot grants	Malawian and Tanzanian researchers, policymakers, and providers	Pilot grant funding for successful applicants	Number of applications for pilot grants Number of pilot grants awarded Number of scientific posters/presentations Number of scientific publications Number of subsequent external grant submissions and grant awards for pilot grantees
5	“Mentor the mentors”	Senior trainees	Mentoring short course (developing syllabi for implementation science and mental health, interactive in-class activities, other approaches to engaging students Monthly calls with SHARP faculty Co-instructor responsibilities for Aim 1 courses in years 4 and 5	Number of Senior Trainees that participate in the program Number of courses Attendance at courses Number of mentees taken on by the cohort of Senior Trainees Course evaluations of Short Courses co-instructed by Senior Trainees

#### Short courses

The success of the introductory and advanced short courses are measured by coverage and knowledge gained. Coverage is the extent to which participants who should be receiving the benefits of an intervention actually do so [42]. Coverage of the introductory and advanced short courses will be measured by the number of courses offered and the number of attendees present in the morning and afternoon during each day of the course. Participant knowledge regarding the subject matter of each course will be measured through a pre-post test administered on the first and last days of each course. In line with knowledge measurement from short course for physicians and nurses, knowledge tests will be developed by course creators and specific to the content of each course [43, 44]. Each test will comprise true/false, multiple choice, and short answer questions. For example, questions for the introduction to implementation science short course knowledge test include: In no more than one sentence, describe what is meant by each of the following types of implementation strategies: (a) discrete, (b) multi-faceted, (c) blended; In no more than one sentence, describe the difference between a Hybrid Type 1 and Hybrid Type 3 design. Short course participants will also complete a course satisfaction evaluation with the goal of measuring course appropriateness that will be used to tailor content for future courses [45].

#### Multifaceted dialogue

The coverage of the multifaceted dialogue platform will be measured though the number of participants attending the Malawi Mental Health Research and Practice Development Conference, the number of webinars, attendance at webinars including new and returning participants, the number or journal clubs, attendance at journal clubs including new and returning participants, and the number of participant posts to the community forum. In addition to coverage, SHARP will also measures the number of accepted posters and presentations to the Malawi Mental Health Research and Practice Development Conference. A survey specific to course content will be administered after each webinar and journal club in order to measure participant satisfaction with the goal of tailoring future webinars.

#### On-the-job training

Crisp et al. [46] recommend several approaches for measuring capacity building efforts for bottom-up organizational approaches that resonate with SHARP’s components. This includes measuring professional development as well as the generation and implementation of ideas [46]. The reach of the on-the-job training program will be measured by the number of admitted participants as well as the number of applications received. The impact of this component will be measure by the number of accepted scientific posters, presentations, and publications by trainees.

#### Pilot grants

The reach of the pilot grants will similarly be measured through the number of proposal applications and the number of funded proposals. We will measure the impact of this component by the number pilot grantee accepted scientific posters and presentations, publications, and the number of subsequent grant submissions and awards.

#### “Mentor the mentors”

Similar to the short courses, the reach of the “mentor the mentors” component will be measured by the number of senior trainees that participate in the program. The coverage of the component will be measured by the number of courses and offered and the number of attendees at each course. We will measure the impact of the component by the number of mentees each participant takes on, as well as their course satisfaction evaluations from their short course co-instruction.

### Program status

Table 2 also provides an overview of current enrollment by component.

#### Short courses

Members of SHARP’s introductory short course Cohort #1 were admitted in March and April of 2018. After a 2-month recruitment period, we admitted 21 applicants from a pool of 31. All 21 applicants received the full introductory short course dose in June of 2018 (five evidence based mental health lectures and five introduction to implementation science lectures). Pre and post knowledge test scores increased for both evidence based mental health knowledge (10% increase), and implementation science knowledge (28% increase). Evaluations for the introductory short courses were high (averaging 3.6/4) and participants noted implementation frameworks as both the best and most challenging short course component. Short course Cohort #2 members were admitted in April and May of 2019. After a 2 month recruitment period, 16 participants were admitted from a pool of 48. All 16 participants received the full introductory short course dose in June of 2019. Similar to the prior cohort, Cohort #2’s evidence based mental health knowledge increased by 6% and implementation science knowledge increased by 29%. Feedback regarding difficulties in understanding implementation theories and frameworks were updated to feature fewer frameworks and provide more real world examples of their application. Course evaluations were stronger in 2019 achieving a 3.7/4 while retaining implementation theories and frameworks as the most useful component.

The 2018 introductory short courses focused on introduction to implementation science were led by a Malawian researcher (study author VM) while auxiliary lectures were delivered by one American researcher (study author CA), one Malawian researcher, and one Malawian NGO worker. 2018 evidence based mental health lectures were facilitated by one American researcher (study author BG). The 2019 introductory short courses were again led by the same Malawian researcher. Additional introduction to implementation science lectures were delivered by the same American researcher and Malawian NGO worker from 2018 but also included lectures from three Tanzanian researchers. The 2019 evidence based mental health lectures were facilitated by a Malawian researcher/mental health practitioner. The majority of short course facilitators constitutes a capacity building effort in itself. Relatively few programs like SHARP exist in Malawi and Tanzania and providing local researchers, NGO workers, and practitioners a platform to develop implementation science and mental health curricula, prepare educational materials, deliver lectures, and evaluate such efforts helps to lay the groundwork for similar courses to continue after the study period.

#### Multifaceted dialogue

The first journal club was also facilitated by partners at UNC-Project Malawi in June 2018 to a total of nine participants, 6 of which were part of the Introductory short course Cohort #1. The article focused broadly on the state of global mental health [47]. The second journal club was run in January 2019 in partnership with the Malawi College of Medicine to a total of 17 participants and explored implementation research in global health at large [48]. The third journal, facilitated by Dignitas International in southern Malawi to 15 participants, took place in February 2019 and discussed task shifting approaches to mental health service integration [49]. SHARP was unable to deliver its final year 1 journal club before June 2019. In May 2019, SHARP delivered its first webinar to a group of 13 Malawian and Tanzanian mental health researchers. The Webinar was led by Dr. Byron J. Powell of UNC-Chapel Hill and focused on implementation theories and frameworks. The community forum is also planned to launch in 2019.

#### On-the-job training

On-the-job training is planned for 2019.

#### Pilot grants

The 2018 RFP for the pilot grants was released in June and close at the end of July. 10 applications were solicited from pairs of researchers/policymakers from which 4 were selected for funding. 3 of the funded proposals emanated from Malawi (2 from the Southern Region and 1 from the Northern Region), and the 4th funded study comes from Dar es Salaam, Tanzania. Funded proposals focus largely on qualitative designs that identify barriers to implementation of evidence based mental health interventions, as well as hybrid designs that measure the impact of implementation strategies on both implementation and clinical outcomes.

## Conclusions

The SHARP Capacity Building Program aims to target the roots of the mental health research and treatment gap in Malawi and Tanzania by enhancing the capacity of researchers, policymakers, and providers. SHARP’s multi-pronged approach will tailor capacity building components to the various needs, goals, and positions of Malawian and Tanzanian researchers, policymakers, and providers. SHARP’s introductory short courses will train a larger, broader cadre of individuals to facilitate dialogue between researchers, policymakers, and providers. The grant writing advanced short course module look to provide content to those interested in obtaining external funding and conducting their own research. SHARP’s “mentor the mentors” component will focus on those positioned to mentor others. Among those poised to deliver or manage evidence based mental health services in the field, SHARP will offer on-the-job training positions on its scale-up study.

While the five components may mitigate the treatment gap on their own, careful timing of the components will allow for synergy between them as well. In order to increase researcher–policymaker dialogue and to produce more policy-centered research, the pilot grant RFPs will be released shortly after the short courses to allow the cohort of researchers, policymakers, and providers to apply their knowledge, connections, and skills. The researcher/policymaker teams whose pilot grants are funded will go on to work closely with SHARP faculty mentors and gain the opportunity to disseminate their protocols and findings at the annual Mental Health Conference. The results from the pilot grants may also be used as preliminary data for the researcher/policymaker pair to use in subsequent grant applications further increasing research capacity in the region. Knowledge and skills will also built though another synergistic pathway when members of introductory short course cohorts apply to, and are selected for, the advanced short course cohorts, the on-the-job training program, or the “mentor the mentors” program as senior trainees.

The SHARP Capacity Building Program is in a unique position to deliver and evaluate these components over the 5-year program period. Given the widespread lack of scaled-up evidence based mental health interventions, results hold meaningful implications for a model of capacity building that could be replicated in other LMICs. If impactful, the SHARP capacity building components could be used to sustainably increase researcher, policymaker, and provider knowledge, skills, and mentorship capabilities regarding evidence-based mental health interventions.

## Data Availability

The datasets generated and/or analyzed during the current study are not publicly available given their focus on small scale, program specific, process outcomes but are available from the corresponding author on reasonable request.
